# Quantifying Titanium Exposure in Lung Tissues: A Novel Laser‐Induced Breakdown Spectroscopy Elemental Imaging‐Based Analytical Framework for Biomedical Applications

**DOI:** 10.1002/smsc.202300307

**Published:** 2024-03-03

**Authors:** Vincent Gardette, Lucie Sancey, Marine Leprince, Laurent Gaté, Frederic Cosnier, Carole Seidel, Sarah Valentino, Frederic Pelascini, Jean‐Luc Coll, Michel Péoc'h, Virginie Scolan, François Paysant, Vincent Bonneterre, Christophe Dujardin, Benoit Busser, Vincent Motto‐Ros

**Affiliations:** ^1^ Université de Lyon Université Claude Bernard Lyon 1 CNRS Institut Lumière Matière Villeurbanne 69100 France; ^2^ Univ. Grenoble Alpes INSERM U1209 CNRS 5309 Institute for Advanced Biosciences Grenoble 38000 France; ^3^ Institut national de recherche et de sécurité pour la prévention des accidents du travail et des maladies professionnelles (INRS) Département Toxicologie et Biométrologie Vandoeuvre‐lès‐Nancy 54519 France; ^4^ CETIM Illkirch‐Graffenstaden 67402 France; ^5^ University Hospital of Grenoble Alpes Grenoble 38000 France; ^6^ Univ. Grenoble Alpes CNRS Grenoble INP TIMC‐IMAG Grenoble 38000 France; ^7^ Institut Universitaire de France Paris 75000 France

**Keywords:** elemental quantification, environmental and occupational hazards, laser‐induced breakdown spectroscopy, lung tissues, titanium exposure

## Abstract

Occupational and environmental exposures, particularly those related to urban and suburban atmospheres, are increasingly linked to a range of pulmonary diseases. While diagnostic methods for these diseases are well established, analytical tools for assessing elemental contamination in lung tissue remain underutilized. This study introduces a novel framework based on laser‐induced breakdown spectroscopy (LIBS) for the in situ quantification of elemental titanium (Ti) in lung tissues from both animal models and human specimens. Rigorous validation is conducted using animal models exposed to TiO_2_ P25 nanoparticles and a comparative analysis with inductively coupled plasma mass spectrometry. The novel quantitative metric $S_{\text{value}}$ demonstrates robust correlation with elemental concentrations, expanding LIBS utility to volumetric organ analysis. This validated methodology is subsequently applied to human lung specimens preserved in paraffin. The research holds significant promise as a diagnostic tool for assessing exposure levels to environmental or occupational hazards, thereby offering valuable contributions to the fields of toxicology and respiratory medicine.

## Graphical abstract

Herein, a novel analytical framework leveraging laser‐induced breakdown spectroscopy for quantifying titanium in lung tissues is introduced. Presenting a robust correlation with elemental concentrations, the method offers a significant advancement in volumetric organ analysis. The approach's efficacy is demonstrated through meticulous biomedical applications, providing a foundational technique for future elemental imaging research or clinical investigations.

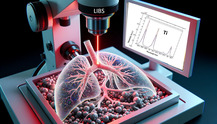

## 1 Introduction

An increasing number of pathologies are being linked to exposure to metal micro‐ and nanoparticles.^[^ ^1^ ^]^ While inhalation of these metallic particles is implicated in a range of lung diseases, such as sarcoidosis, pneumoconiosis, and pulmonary fibrosis, the biological mechanisms underlying these idiopathic conditions remain largely elusive.^[^ ^2^ ^]^

To deepen the understanding of such pathologies and assess the sources of exposure, whether occupational or environmental, clinicians require robust methods for in situ quantitative identification of metals in human tissue samples. Recent years have seen the emergence of various techniques for imaging particle distribution, including laser ablation inductively coupled plasma mass spectrometry (ICP‐MS),^[^ ^3^, ^4^ ^]^ synchrotron radiation micro‐X‐ray fluorescence microanalysis, and particle‐induced X‐ray emission,^[^ ^5^ ^]^ either as standalone methods or in combination.^[^ ^6^ ^]^ However, these techniques often prove impractical for routine clinical applications due to operational constraints.

Recent studies have explored the application of scanning electron microscopy with energy‐dispersive spectroscopy (SEM‐EDS),^[^ ^7^ ^]^ conducted on thinly sliced tissues placed on a conductive substrate. While this technique provides valuable insights by visualizing particles and directly characterizing their elemental composition, it has several limitations. First, SEM‐EDS offers a restricted analysis area, typically covering only 200 × 200 μm per hour of measurement, thereby proving inadequate for analyzing entire biopsy samples, which often span several cm^2^. Second, this method falls short in detecting light elements like beryllium or lithium, which are implicated in diseases such as chronic beryllium disease. Finally, while SEM‐EDS is suitable for detecting particles with dimensions around 1 μm, it is ineffective for resolving smaller particles. Transmission electron microscopy could potentially detect nanoparticles smaller than 1 μm, but this comes at the cost of complex sample preparation and a significantly reduced analysis area (≈10 × 10 μm). Consequently, these methodologies are not amenable to routine clinical diagnosis, which necessitates imaging and quantifying several metals over much larger biopsy areas.

Laser‐induced breakdown spectroscopy (LIBS) emerges as a highly promising technique for both imaging and quantitative analysis of metal particles in biological tissues, satisfying biomedical performance and instrumentation criteria.^[^ ^8^ ^]^ This method boasts several compelling advantages: rapid data acquisition (operable up to the kHz regime), comprehensive elemental detection (capable of identifying all periodic table elements), detection limits typically within the parts‐per‐million (ppm) range (element‐dependent), and functionality in ambient conditions. Importantly, being an optically based method, LIBS is seamlessly compatible with optical microscopy, a staple in hospital settings for histopathological examinations. LIBS microimaging appears as a robust tool for tissue analysis, but so far studies were mainly focused on endogenous elements.^[^ ^9^, ^10^ ^]^ Our research group has already demonstrated successful metal‐nanoparticle imaging in biological tissues using LIBS to study the elemental distribution and toxicology of gadolinium‐based nanoparticles^[^ ^11^, ^12^ ^]^ as well as the first LIBS elemental analysis of human formalin‐fixed, paraffin‐embedded (FFPE) biopsies.^[^ ^13^, ^14^ ^]^ In prior studies, tissue specimens from rodents were either fixed and embedded in epoxy resin to enhance spatial resolution and measurement repeatability or were simply flash‐frozen, sectioned, and analyzed.^[^ ^12^ ^]^ Additional work from our group has also used LIBS to investigate the tumor‐targeting properties of cerium/lanthanum nanoparticles in preclinical settings.^[^ ^15^ ^]^

Among various metallic nanoparticles, titanium dioxide (TiO_2_) is especially important due to its widespread use in industries, such as paints, coatings, plastics, paper, and food production. This ubiquity increases the risk of occupational and environmental exposure.^[^ ^16^ ^]^ In addition to its known toxicities, including genotoxic and carcinogenic potentials, the inhalation of TiO_2_ particles has been linked to adverse pulmonary outcomes, notably inflammation and fibrotic responses.^[^ ^17^ ^]^ Regulatory authorities, including the International Agency for Research on Cancer (IARC) and the U.S. National Institute for Occupational Safety and Health (NIOSH), have acknowledged these health risks, advocating for continued research to refine safety standards and regulations.^[^ ^18^, ^19^ ^]^ Despite TiO_2_'s common use, detailed investigations into the respiratory implications of TiO_2_ particle inhalation and the consequent health effects remain limited.

In this study, we introduce an innovative methodology for calibrating and quantifying metal nanoparticles in soft biological samples using LIBS imaging. Specifically, we target, image, and quantify titanium (Ti) in FFPE lung specimens from both animals and humans. The FFPE preparation of tissues aligns with standard medical protocols. Mastering laser‐induced plasma generation on such soft substrates presents significant challenges, notably the shock wave effect that can adversely affect the surface near the ablation site.^[^ ^12^ ^]^ This consideration is critical when imaging biological specimens at high speeds (100 Hz), a cornerstone of our strategy to achieve both rapid and reliable elemental analyses. For calibration, we used rat lung tissues exposed to well‐characterized TiO_2_ P25 nanostructured aerosols (NSA) via controlled inhalation procedures. Our method yielded a robust linear correlation between LIBS signal intensities for Ti and levels of exposure, facilitating the creation of a reliable calibration curve. Leveraging our validated approach, we mapped Ti concentrations in human lung specimens, taking a crucial initial step toward the future clinical application of LIBS as a rapid and versatile diagnostic tool.

## 2 Results and Discussion

### 2.1 Definition of the *S*_value_

In clinical settings, accurate localization of exogenous elements within biological tissue is paramount. This necessitates the utilization of histological images obtained from adjacent sections as a referential framework. One primary aim of this elemental imaging study was to generate images depicting endogenous elements, such as phosphorus (P), magnesium, and sodium, in order to reconstruct a tissue architecture that closely mirrors its histological counterpart. Subsequent imaging focuses on the identification of exogenous elements, particularly various metals that may be present in either microparticle or nanoparticle form. The data collected from these imaging techniques can be substantial, posing a challenge for routine clinical application due to the sheer volume of specimens and images to be analyzed. Additionally, the contrast within the images complicates the quantification of exogenous elements, a challenge further exacerbated when examining metal particle distributions in lung tissue, which frequently manifest as isolated pixels. To address this issue, we introduced a quantitative metric, designated as $S_{\text{value}}$. This metric facilitates an easier interpretation by clinicians, enabling the rapid identification of abnormal elemental concentrations. When warranted, clinicians can then scrutinize the images in greater detail to elucidate the spatial distribution of these elements within the tissue.

The challenge in determining a $S_{\text{value}}$ arises from the intricate nature of the samples under investigation: tissues embedded in paraffin. Existing market offerings lack commercial calibration standards adapted to our study, i.e., paraffin‐embedded lung tissue reference materials that possess a homogeneous and known concentration of chemical elements of interest, especially for Ti. Consequently, we adopted a strategy utilizing lung specimens from animals exposed to relevant conditions, thereby establishing a calibration curve specifically for the extraction of a metric $\left(S_{\text{value}}\right)$ for Ti. Our dual objectives encompassed evaluating analytical figures of merit, such as linearity, detection limits, and uncertainties, and validating the hypothesis that an elemental analysis of a lung section could be representative of the entire lung volume.

To compute $S_{\text{value}}$, it is essential to aggregate the significant elemental intensities, denoted as I(Ti), and normalize this sum by the total tissue area $A_{\text{tissue}}$

(1) $$S_{\mathrm{value}}(\mathrm{Ti})=\frac{\sum I(\mathrm{Ti})}{A_{\mathrm{tissue}}}\;$$

This calculation requires to address several challenges such as establishing a procedure to define the tissue area, defining an appropriate methodology to isolate the Ti line intensities and their corresponding uncertainties.

### 2.2 Spectral Characterization and Elemental Mapping of Endogenous and Exogenous Markers in Lung Tissue

**Figure 1:**
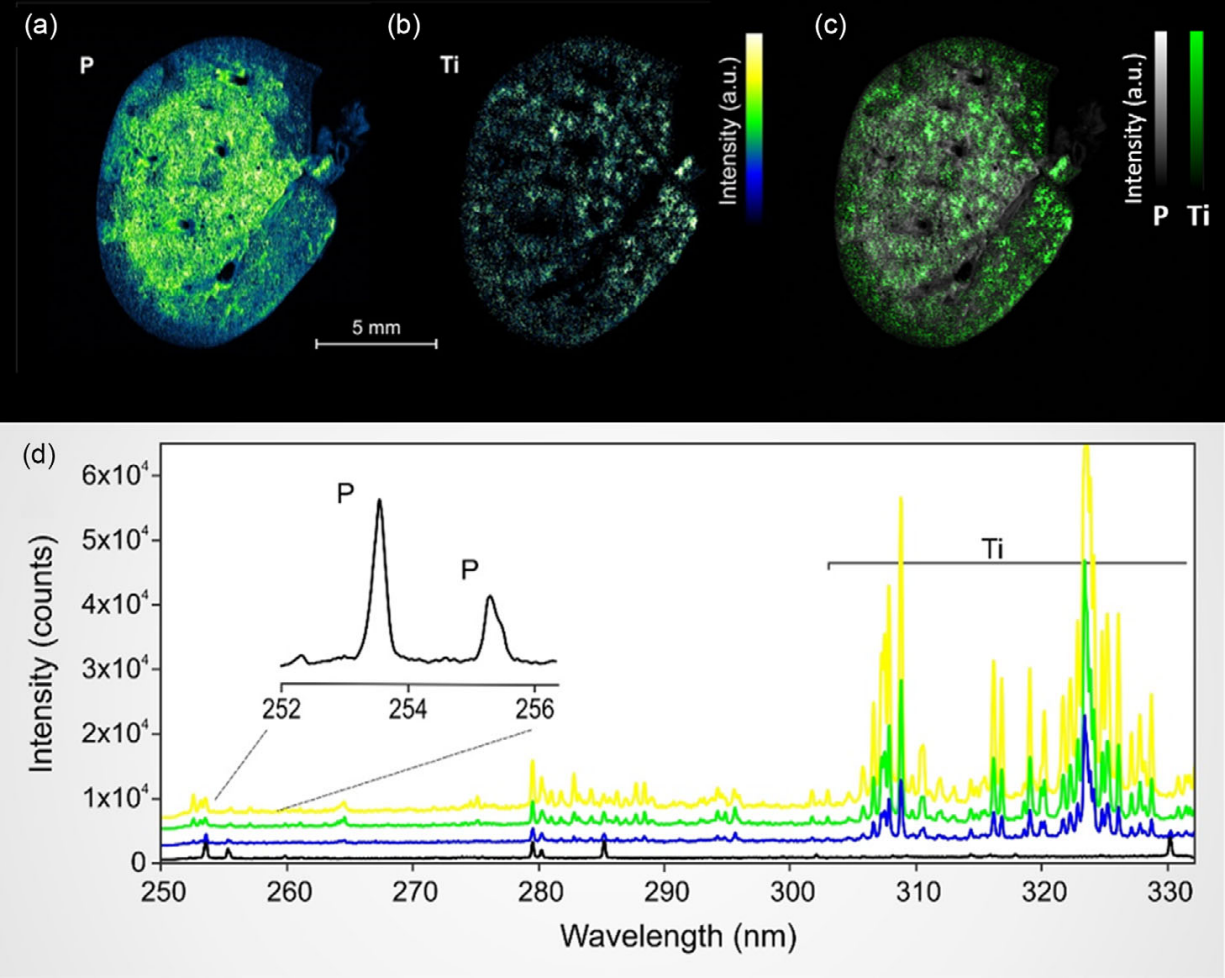
High‐resolution elemental imaging and spectral analysis of P and Ti via LIBS. a) Elemental map revealing the distribution of endogenous P within a representative sample of rat lung tissue. b) Elemental map highlighting the spatial localization of exogenous Ti, derived from inhaled TiO_2_ nanoparticles. Both maps are generated at a granular resolution of 1000 × 1000 pixels, featuring a lateral resolution of 20 μm. c) Merged elemental mapping of P and Ti in rat lung tissue. The image merges elemental maps of endogenous P (depicted in grey) and exogenous Ti (depicted in green), originally presented in a and b). This composite view facilitates the simultaneous interpretation of both elements’ spatial distributions within the tissue sample. d) Representative emission spectra illustrate four distinct levels of titanium intensity, each originating from unique spatial positions within the same tissue sample, as shown in c). The color coding of spectra represents a diversity of titanium intensities, ranging from the absence of titanium (black), through medium intensities (blue and green), to high titanium concentrations (yellow).

In the initial phase of the study, emission lines for P and Ti were selected to avoid spectral interference, defined as the overlapping of emission lines from different elements. Phosphorus, an integral component of DNA and biomembranes, serves as an endogenous marker because its elemental image indirectly maps the overarching architecture of the biological tissue. Conversely, Ti serves as an exogenous marker, specifically denoting the presence of inhaled TiO_2_ nanoparticles within the animal lung tissue. Through the employment of a carefully defined spectral window, we achieved the detection of intense emission lines corresponding to both elements. **Figure** **1**a,b displays representative relative abundance maps for P and Ti obtained after LIBS imaging procedure.

To our knowledge, the megapixel elemental images presented in Figure 1a–c are among the few of their kind generated from biomedical specimens using LIBS. The 20 μm lateral resolution enables a comprehensive characterization of the tissue, and a closer examination of the selected specimen (Figure 1) reveals a porous lung tissue architecture delineated into two phases: a centrally located, phosphorus‐rich region, and a peripherally located region with lower phosphorus concentration. Titanium displayed a largely homogeneous distribution within the lung tissue, as illustrated in Figure 1c. This homogeneity attests to the efficacy of the inhalation exposure procedures employed.^[^ ^20^ ^]^

The spectral window chosen for the spectrometer accommodates multiple emission lines for the elements of interest, including two lines for P and numerous lines for Ti (as shown in Figure 1d), ensuring an accurate detection of the elements of interest. Moreover, this spectral window also encompasses primary or intense emission lines for other elements of interest in the context of inhalation exposure, such as aluminum (Al), copper (Cu), silicon (Si), chromium (Cr), iron (Fe), beryllium (Be), nickel (Ni), and zirconium (Zr). Figure 1d features four emission spectra with different Ti intensities, each shifted along the *y*‐axis for clarity.

Due to its high dynamic range, LIBS emerges as an exceptionally suitable technique for detecting exogenous elements across a broad concentration spectrum—from ppm to tens of percentage points. It is also adapted for detecting all the endogenous elements which are ubiquitous in the tissues. This capability facilitates monitoring of specific elements across six orders of magnitude in concentration, all while maintaining rapid data acquisition and imposing minimal experimental constraints.

### 2.3 From Elemental Imaging to Quantitative Analysis

Upon the acquisition of LIBS elemental maps for abundance quantification, the background correction and surface contamination become critical because it becomes imperative to rigorously correct and control the signal to mitigate potential artifacts. While this issue of contamination is not unique to paraffin‐embedded samples, we employed analytical chemistry‐specific thresholds, namely, the limit of detection (LoD)^[^ ^21^ ^]^ and the limit of quantification (LoQ),^[^ ^22^, ^23^ ^]^ to effectively filter the entire data set.

**Figure 2:**
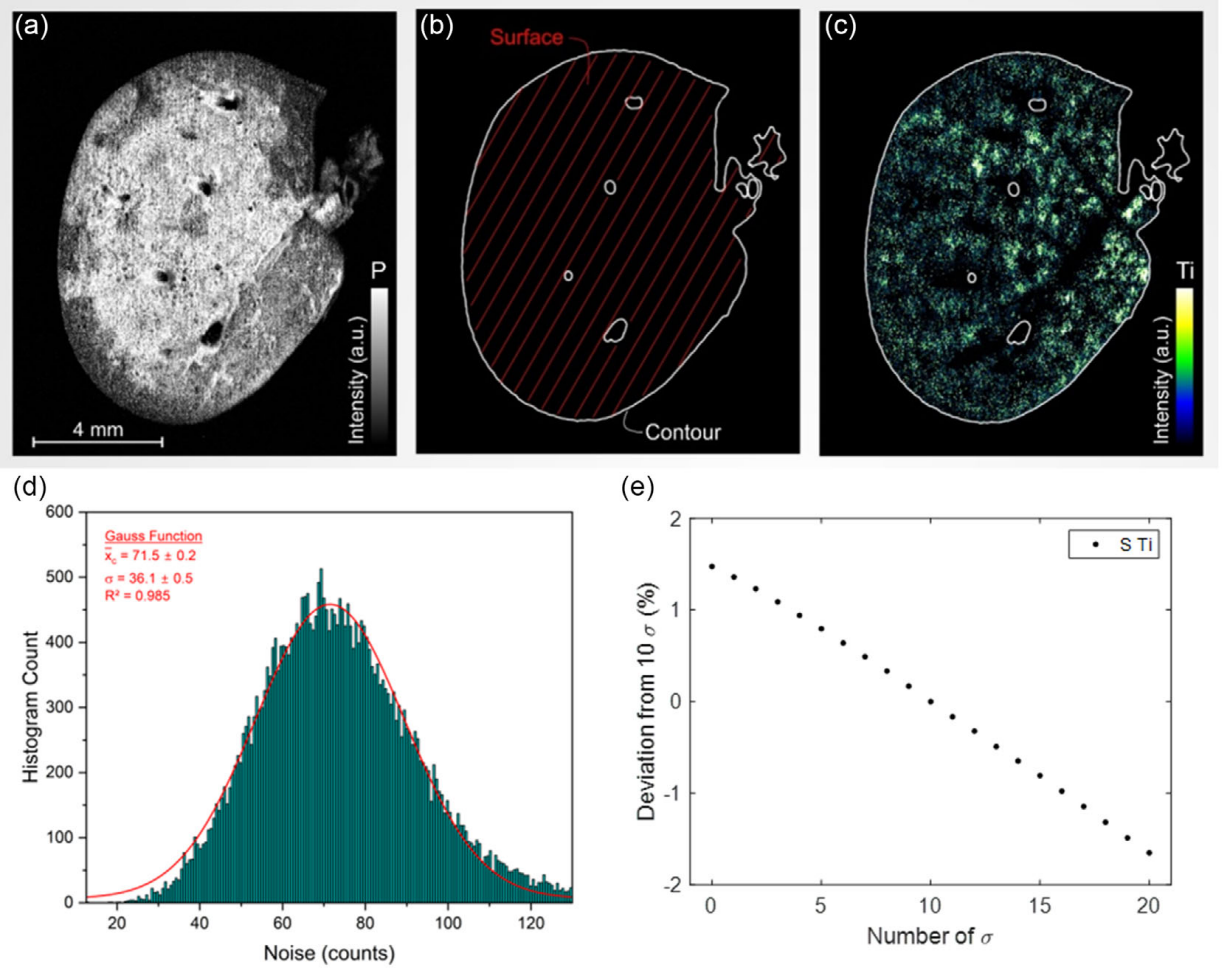
Methodological workflow for the creation of a phosphorus‐based tissue mask in elemental imaging. a) Acquired elemental image emphasizing P content, which serves as a proxy for the biological tissue under examination. b) Application of a statistically determined threshold value for phosphorus to create a mask that isolates pixels specifically associated with tissue. c) Subsequent exclusion of intensity values for target elements (e.g., Ti) that fall outside the boundaries of the established tissue mask. d) Histogram of spectral intensity distributions in the noise‐dominated spectral window of 320–322 nm. The histogram represents the distribution of intensities across more than 32 000 tissue spectra. A Gaussian fit to the data, shown in red, aids in characterizing the inherent noise signal within this specific spectral window, which is deliberately chosen for its absence of elemental emission lines. e) Error quantification of the signal threshold for Ti intensities. $S_{\text{values}}$ from 0 to 20*σ* are compared from the reference value of 10*σ*.

These thresholds are conventionally derived from signal of the noise. Specifically, the LoD functions as a tissue‐specific threshold, while the LoQ operates as a signal threshold. For the purposes of noise extraction, we focused on spectra corresponding to tissue within a 2 nm spectral window, ranging from 320 to 322 nm—a range out but close to the Ti emission line of interest. The noise was determined using a substantial dataset of 32 000 spectra, as illustrated in **Figure** **2**d.

It is noteworthy that the observed noise distribution deviates slightly from a Gaussian fit. This discrepancy is likely attributable to the right tail of the distribution, which may represent a composite of noise and low‐level titanium signals, given titanium's broad emission spectrum featuring numerous lines. Subsequent to the extraction of the noise and its standard deviation, the LoD and LoQ^[^ ^24^ ^]^ are computed as follows

(2) $$\mathrm{LoD}=\;\bar{x_{c}}+3\sigma \;;\;\mathrm{LoQ}=\;\bar{x_{c}}+10\sigma \;$$

where $\bar{x_{c}}$ and *σ* are the averaged noise and its standard deviation, respectively.

As a first analysis step, we delineated the area of interest by constructing a mask derived from the P elemental map, which corresponds to the tissue surface, denoted as $A_{\text{tissue}}$ (Figure 2a,b). The LoD serves as the tissue threshold, facilitating the differentiation between paraffin‐embedded regions and genuine tissue. Specifically, if the P signal surpasses this LoD threshold, the region is classified as tissue. Conversely, signals falling below the LoD are likely attributable to noise fluctuations and are consequently excluded from consideration. To quantify the uncertainty associated with this threshold, we assessed the deviation in $A_{\text{tissue}}$ when altering the standard deviation (*σ*) thresholds from 3*σ* to both 2*σ* and 4*σ*. This sensitivity analysis yielded a surface deviation of ≈6%.

As a second step, a filtration process is applied specifically to the Ti intensities within the tissue mask. The signal threshold for this stage is established at the LoQ, and intensities falling below this cutoff are systematically excluded. As a third step, the sum of all intensities corresponding to all Ti‐positive pixels is then normalized by the tissue area ($A_{\text{tissue}}$). The resultant metric, termed $S_{\text{value}}$, serves as a quantitative indicator of the total Ti content per unit of tissue area and allows for intersample comparability.

In alignment with analytical chemistry standards, a 10*σ* threshold was employed for signal discrimination. It should be noted, however, that variations in the number of *σ* applied for signal thresholding exert a minimal influence on $S_{\text{value}}$, given that nontissue spectra have already been eliminated through the initial tissue‐specific thresholding. Detailed sensitivity analyses regarding the deviation of $S_{\text{value}}$ for different *σ* signal thresholds linked to the Ti accumulation revealed an associated uncertainty margin of 0.89% by comparing the reference $S_{\text{value}}$ to those for ± 3*σ* (Figure 2e).

### 2.4 Calibration Methodology

A cohort of six rat specimens from each exposure group—categorized as low, medium, and high—was subjected to ICP‐MS for the quantification of total Ti mass in the exposed lung tissues. In each animal, the cranial right lung lobe underwent digestion and subsequent ICP‐MS analysis for Ti quantification. Concurrently, the caudal right lung lobe was preserved through formalin fixation and paraffin embedding for LIBS analysis.

**Figure 3:**
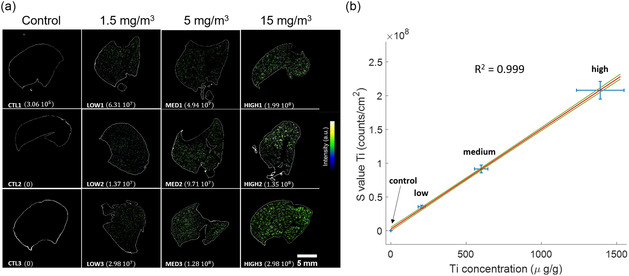
Quantitative analysis and calibration for Ti exposure in rat lung samples. a) Representative LIBS elemental images displaying Ti distribution across various levels of exposure in three rat lungs per condition (CTL, control; LOW, low exposure; MED, medium exposure; HIGH, high exposure; lateral resolution: 50 μm). $S_{\text{value}}$ for each specimen is expressed in counts/cm^2^. b) Calibration curve comparing Ti mass concentrations quantified via ICP‐MS (*x*‐axis, in μg g^−1^) against LIBS signals (*y*‐axis; $S_{\text{value}}$). Confidence intervals are depicted in green, established at a 70% confidence level, to quantify the statistical reliability of the calibration model.

The computational approach delineated earlier for calculating $S_{\text{value}}(\mathrm{Ti})$ was effectively implemented on the set of 12 selected lung samples, facilitated by a fully automated MATLAB code. A comparative analysis was performed between the Ti concentrations obtained through ICP‐MS and the $S_{\text{value}}(\mathrm{Ti})\;$ ascertained via LIBS imaging. This comparison was executed on a subset of three samples from each exposure group, as illustrated in **Figure** **3**.

Despite uniform exposure to TiO_2_ nanoparticles across all exposure groups, heterogeneities were observed in Ti concentrations within individual samples (e.g., variations in $S_{\text{value}}(\mathrm{Ti})\;$ within the low‐exposure cohort, refer to Figure 3a). The calibration curve, depicting $S_{\text{value}}(\mathrm{Ti})$ against ICP‐MS (Ti) concentrations (see Figure 3b), demonstrated remarkable linearity, with a coefficient of determination (*R* ^2^) exceeding 0.999. Importantly, the calibration curve intersects the origin within the bounds of model uncertainties, substantiating the adequacy of the $S_{\mathrm{value}}$ extraction methodology.

Derived from the calibration curve, both the LoD and the LoQ can be computed utilizing the robustness of the curve fit^[^ ^22^, ^23^ ^]^

(3) $$\mathrm{LoD}=3.3\frac{\sigma_{\mathrm{fit}}}{m}\;;\;\mathrm{LoQ}=10\frac{\sigma_{\mathrm{fit}}}{m}$$

Here, $\sigma_{\text{fit}}$ and *m* denote the standard error of the fit and the slope of the calibration curve, respectively. This innovative methodology achieves sub‐ppm sensitivity, with a LoD registering at 0.05 μg g^−1^ and a LoQ at 0.16 μg g^−1^.

The calibration curve's exceptional linearity yields two pivotal insights: i) The $S_{\text{value}}(\mathrm{Ti})$ obtained through LIBS imaging strongly correlates with the total Ti content measured via ICP‐MS. This suggests that $S_{\text{value}}\;$ serves as a robust quantitative indicator for total Ti content per unit mass of tissue; and ii) $S_{\text{value}}(\mathrm{Ti})\;$ is proportional to the levels of exposure to inhaled TiO_2_ nanoparticles in rats, affirming the robustness of LIBS imaging for evaluating respiratory exposure to xenobiotic substances.

While ICP‐MS provides volumetric measurements for entire lung lobes, LIBS remains a surface‐sensitive technique with a depth of analysis approximating 10 μm in paraffin‐embedded samples. The observed positive correlation between surface‐level Ti signals obtained via LIBS and global Ti mass quantified by ICP‐MS substantiates that LIBS surface measurements may serve as viable proxies for volumetric lung content. This is contingent upon a homogeneous distribution of the particles of interest—specifically, TiO_2_ nanoparticles—induced by the exposure. In the current study, the similarity in Ti content between cranial and caudal lung lobes further corroborates that the exposure protocol ensured a uniform distribution of inhaled TiO_2_ nanoparticles throughout the lung volume.

### 2.5 Influence of Lateral Resolution on Analytical Efficacy

The high‐definition elemental image illustrated in Figure 1 demands ≈3 h for comprehensive analysis, given that the used LIBS instrumentation, operating at 100 Hz, can record up to 360 000 spectra per hour. While this time investment aligns well with academic research requirements, it presents logistical challenges for routine clinical applications. Notably, the acquisition time is inherently dependent on two factors: the lateral resolution and the surface area of the sample under investigation. For human specimens, which can span up to 6 cm^2^, the time commitment becomes prohibitive, requiring around 40 min per cm^2^ at the lowest feasible lateral resolution of 20 μm.

To reconcile the need for rapid analysis with analytical rigor, we scrutinized the impact of diminishing lateral resolution—defined as the step size or inter‐laser‐shot distance—on the $S_{\text{value}}$ metric. Given that exogenous elements in lung tissues often manifest as discrete particles (isolated pixels), excessive degradation of the resolution could compromise the elemental image accuracy. To model this, we employed the original high‐definition image from Figure 1 to artificially generate submaps with reduced resolution via random pixel selection. For example, within a 4 × 4 pixel square (initially captured at a 20 μm step size), a single pixel was randomly chosen to reconstruct an image at an 80 μm resolution. This process was iteratively applied to generate 100 degraded images for each examined step size, ranging from 20 to 200 μm, and the corresponding $S_{\text{value}}(\mathrm{Ti})\;$ was computed for each.

**Figure 4:**
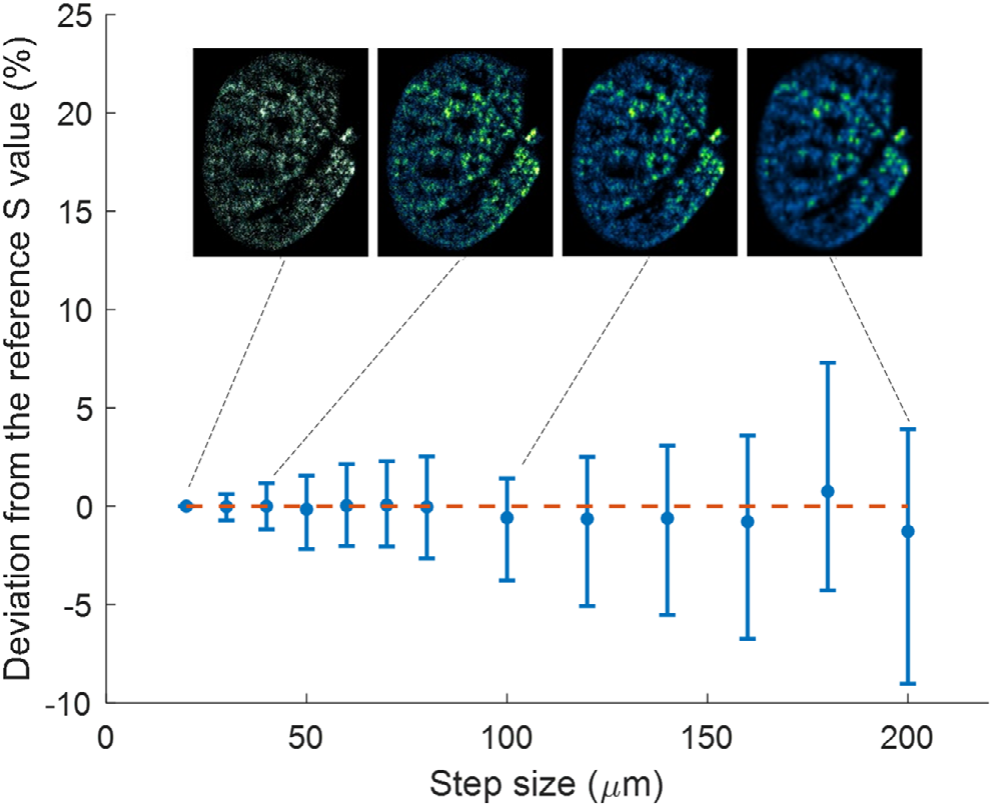
Impact of step size on Ti signal intensity and variability. The figure illustrates the extrapolation of derived $S_{\text{value}}(\mathrm{Ti})\;$metrics across the entire sampled area as a function of lateral resolution (step size) between two laser shots. For each resolution setting, both the mean and standard deviation of $S_{\text{value}}(\mathrm{Ti})$are displayed.

As illustrated in **Figure** **4**, the mean $S_{\text{value}}(\mathrm{Ti})\;$ for degraded images closely approximates the reference $S_{\text{value}}(\mathrm{Ti})\;$ derived from high‐resolution imaging. However, statistical uncertainties, represented by standard deviations, increase with resolution deterioration, peaking at ≈10% for a tenfold reduction in resolution (i.e., 200 μm). This deterioration in resolution, however, leads to a significant reduction in analysis time by a factor of 100. Importantly, a moderate resolution shift from 20 to 50 μm decreased the acquisition time from 3 h to 25 min while maintaining a standard deviation below 5%. Moreover, the elemental distribution of Ti remained consistent at this 50 μm resolution, mirroring the homogeneity observed in the original high‐definition image. Consequently, a 50 μm resolution emerges as an optimal compromise between analytical speed and confidence in quantitative assessments. Note that recent technologies have improved the laser repetition rate up to the kHz, and kHz LIBS imaging has been demonstrated.^[^ ^25^ ^]^

### 2.6 Quantification of Analytical Uncertainties

In Figure 3b, the error bars along the *x*‐axis signify the standard deviations derived from ICP‐MS measurements within identical exposure groups. The composite error on the *y*‐axis, denoted as $\sigma_{\text{tot}}$, is an aggregate of uncertainties emanating from various stages of $S_{\text{value}}$ computation. This is mathematically represented as

(4) $$\sigma_{\mathrm{tot}}=\sqrt{\sigma_{\Delta x}^{2}+\sigma_{\mathrm{surf}}^{2}+\sigma_{\sum I}^{2}+\sigma_{\mathrm{CC}}^{2}}\;$$

Here, $\sigma_{\Delta x}$, $\sigma_{\text{surf}}$, $\sigma_{\sum I}$, and $\sigma_{\text{CC}}$ correspond to the uncertainties associated with lateral resolution (from sample shown Figure 1), tissue thresholding, signal thresholding for intensity summation, and the confidence curve at the median value, respectively. These individual error components are quantified as 1.8%, 6%, 0.89%, and 2.2%, cumulatively yielding a total error of 6.7%.

### 2.7 Application to Human FFPE Specimens

Having established a Ti calibration curve via LIBS quantitative analysis on animal FFPE lung tissues exposed to varying concentrations of TiO_2_ P25 nanoparticles, we proceeded to adapt this novel methodological framework for quantifying elemental Ti content in autopsied human FFPE lung samples.

**Figure 5:**
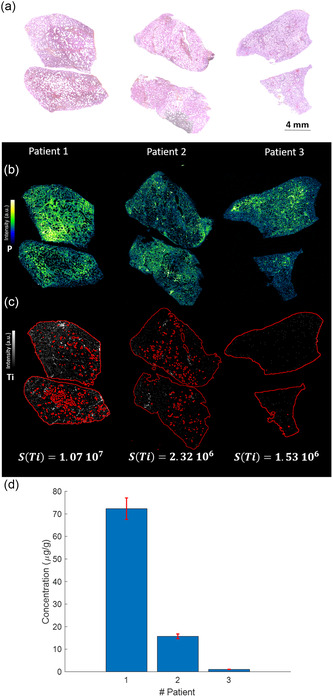
Quantitative LIBS analysis of Ti in human FFPE lung specimens. a) Hematoxylin and Eosin (H&E) staining of lung tissues from three autopsied individuals (Patients 1, 2, and 3), derived from 5 μm thick tissue sections. b) Elemental LIBS image of P and c) Ti in adjacent paraffin blocks. The $S_{\text{value}}$ expressed in counts/cm^2^ are indicated for each case. d) Quantitative analysis of Ti concentrations, calculated using the established LIBS calibration methodology.

**Figure** **5**a,b provide histological and phosphorus elemental images, respectively, to elucidate the tissue architecture of the three human lung specimens. Subsequently, the LIBS‐derived elemental images for Ti are displayed in Figure 5c.

After employing the calibration curve illustrated in Figure 3b, the calculated yielded $S_{\text{value}}(\mathrm{Ti})\;$ corresponding Ti concentrations for these patients measured at 72.3, 15.7, and 1.03 μg g^−1^. Notably, these observed concentrations align well with literature, wherein the typical range for human lung Ti content spans between 0.1 and 200 μg g^−1^.^[^ ^26^, ^27^, ^28^ ^]^ Drawing upon the empirical data obtained from controlled inhalation exposure studies in animals (Figure 3b), the observed Ti concentrations in the three human lung specimens can be interpretatively aligned with distinct levels of exposure. Specifically, the elevated Ti content in Patient 1's lungs suggests a high level of exposure, while the moderate and minimal concentrations in Patients 2 and 3, respectively, could be indicative of medium and low exposure levels. The multielemental capability of LIBS imaging was also leveraged to generate elemental images for both endogenous elements, such as sodium and magnesium, and exogenous elements, including silicon and aluminum (Figure S1, Supporting Information). The LIBS‐based analytical framework presented herein holds significant promise as a diagnostic tool for medical pathologists and clinicians. Specifically, it offers the capability to directly quantify concentrations of specific chemical elements—particularly metals—in lung tissue biopsies. This could serve as a valuable adjunct in assessing patient exposure levels to environmental or occupational hazards.

## 3 Conclusion

In the present study, we have successfully formulated a novel LIBS‐based analytical method for elemental mapping of Ti in both animal and human lung tissues. Conceived to augment existing histological analyses in clinical practice, this methodology paves the way for future applications of LIBS in quantifying foreign exogenous exposure in human biological samples.

To rigorously assess the method's efficacy being user decision free, initial applications were confined to the quantification of a singular chemical element—titanium—in animal lung tissues exposed to TiO_2_ P25 nanoparticles. These served as pertinent proxies for the anticipated human applications. The introduction of the $S_{\text{value}}$ metric, applicable across various elements, offers a reliable means of correlating elemental concentrations in biological tissues. Furthermore, the entire methodology is predicated on the principles of analytical chemistry, specifically focusing on signal treatment techniques such as LoD and LoQ. The process is fully automated, thereby mitigating the potential for errors attributable to human decision‐making.

A comparative analysis with ICP‐MS underscored the representativeness of LIBS surface measurements for entire organ volumes, at least within the context of this study. Although the focus here was Ti quantification, the outcomes of this research provide a foundational proof‐of‐concept for the broader applicability of LIBS in in situ quantification of elemental exposure levels in biomedical specimens.

Given rising concerns over inhalation of aerosols and respirable particulates, this work holds significant implications for the fields of toxicology and respiratory medicine. The methodology offers the capability for imaging a diverse array of xenobiotic substances, each with their own intrinsic sensitivity thresholds, thereby promising a wide scope for future research and clinical applications

## 4 Experimental Section

4.1

4.1.1

##### Animal Specimens

All animal experiments were executed in strict compliance with the European Union Directive 2010/63/EU and French legislation governing the ethical treatment of animals for research purposes. The study was conducted in a laboratory accredited by the French Ministry of Agriculture (Accreditation No. D54–547‐10) and received full approval from both the local Ethics Committee and the French Ministry for Research and Higher Education (Authorization No. APAFIS#10 052). *To adhere to the 3 Rs principle, particularly with the aim of reducing animal use, we repurposed rat lung samples initially utilized in the EU Horizon 2020 project SmartNanoTox, under Grant Agreement No. 686 098.*

Animal exposure to NSA was performed in 2018 in accordance with OECD test guideline no. 403.^[^ ^16^ ^]^ Female Sprague–Dawley rats, aged 7 weeks, were procured from Janvier Labs, France. They were housed under controlled environmental conditions: relative humidity at 55 ± 10%, temperature at 22 ± 2 °C, and a 12 h light/dark cycle. Access to water and a standard laboratory animal diet (A04 Safe diet) was unrestricted.

Prior to exposure, the rats underwent a 2 week acclimatization period in restraining tubes to prepare them for nose‐only exposure to TiO_2_ P25 NSA (TiO_2_ NSA). Subsequently, rats aged 10 weeks were exposed to either filtered air or TiO_2_ NSA at a concentration of 15 mg m^−3^ for 6 h per day, 5 d per week, over a 2 week period. To create three distinct exposure groups with equivalent 6 h concentrations (1.5, 5, and 15 mg m^−3^), we modulated the exposure time to TiO_2_ NSA (36, 120, and 360 min, respectively). This was based on the assumption of a consistent time‐dependent lung deposition of the NSA, as per the Concentration × Time (*C* × *t*) protocol outlined.^[^ ^29^ ^]^

The TiO_2_ NSA was generated by a rotating brush aerosol generator (RBG1000, PALAS, Karlsruhe, Germany) from a mixed‐phase nanocrystalline TiO_2_ powder (AeroxideP25, Evonik), composed of 80% anatase and 20% rutile crystallites. The average primary particle diameter was 21.5 ± 7.2 nm. Comprehensive aerosol monitoring and characterization were conducted in alignment with a previously established characterization strategy.^[^ ^20^ ^]^ Specifically, the aerosol exhibited a count median aerodynamic diameter of 0.31 μm with a geometric standard deviation of 1.72. The mass median aerodynamic diameter was 1.56 μm, and the aerosol number concentration measured 51 000 ± 17 000 particles/cm^3^.^[^ ^30^ ^]^

Animals were euthanized 3 d postexposure. Lung lobes were harvested and cryopreserved at −80 °C until subsequent analysis. Titanium content in the cranial right lung lobes was quantified using ICP‐MS. The caudal right lung lobes were fixed in neutral buffered formalin (Sigma–Aldrich) and, following dehydration, were embedded in paraffin blocks for LIBS analysis. Detailed protocols for sample collection, preparation, and analysis have been comprehensively described in prior publications.^[^ ^31^, ^32^, ^33^, ^34^ ^]^

**Table** **1** provides a comprehensive summary of the actual atmospheric concentrations to which each exposure group was subjected, as well as the quantities of retained Ti within the lung tissue, measured 3 d postexposure cessation.

**Table 1:** Summary of actual TiO_2_ atmospheric mass concentrations and retained Ti doses in lung tissue

Exposure group	Number of samples	Target concentration [mg m^−3^]a)	Actual concentration [mg m^−3^]b)	Ti retained dose [μg] (Ti/lung)c)
Control	6	0	0	0.2 ± 0.1
Low	6	1.5	2.22 ± 0.64	209 ± 59
Medium	6	5	5.04 ± 1.30	554 ± 142
High	6	15	15.3 ± 3.54	1477 ± 213

a) Six hour equivalent concentrations for each exposure group;

b) Mean ± standard deviation values for TiO_2_ atmospheric concentrations, derived from 1, 2, and 4 daily gravimetric samplings for the low, medium, and high concentration groups, respectively;

c) Mean ± standard deviation of retained Ti doses, with a sample size of *n* = 10 per group.

##### Human Specimens

Three human lung specimens were procured from forensic autopsies conducted in 2019 by clinicians affiliated with the Forensic Department of Grenoble University Hospital (CHUGA). These autopsies were executed under the auspices of the research protocol designated as “controLIBS”, which received approval from the District Judicial Attorney of Grenoble in April 2018. The protocol and subsequent autopsy studies adhered rigorously to the guidelines stipulated by the Clinical Research Unit of CHUGA. The research was conducted within the regulatory framework, bearing the INDS (formerly HDH) registration number no. I07103007192019 and the MR0004 conformity statement no. 2 205 066 v.0. Postautopsy, lung tissues were subjected to formalin fixation and paraffin embedding, in accordance with the standard histological procedures of CHUGA. Inclusion criteria for the lung samples in the controLIBS study were stringently applied; only specimens deemed normal by the institution's legal pathologist were included. Specifically, samples manifesting no standard lung histopathological features such as emphysema, fibrosis, hemorrhage, tumors, or necrosis were considered eligible.

##### LIBS Elemental Imaging

LIBS was performed directly on FFPE blocks, requiring only surface refreshing as a preparatory step. The technical configuration employed has been comprehensively delineated in previous publications.^[^ ^11^, ^13^ ^]^ In summary, a neodymium‐doped yttrium aluminum garnet (Nd:YAG) laser (Centurion, Quantel Laser) was utilized, operating at the fundamental wavelength with a 100 Hz repetition rate and a pulse energy set at 2.5 mJ. Laser pulses were focused onto the sample surface via a 15x magnification objective (LMM‐15X‐P01, Thorlabs). Samples were methodically traversed on a motorized *XYZ* platform, allowing pixel‐by‐pixel scanning. The intershot distance was adjustable, ranging from 10 to 100 μm. Both laser pulse energy and focal distance relative to the sample surface were stringently monitored throughout the experiments. LIBS measurements were conducted under ambient conditions: room temperature and atmospheric pressure, with an argon flow of 1.0 L min^−1^ aimed at the plasma region for confinement and control. Emitted plasma radiation was captured by a lens‐fiber system connected to a Czerny–Turner spectrometer (Shamrock 500, Andor Technology), equipped with an intensified charge‐coupled device (ICCD) camera (Istar, Andor Technology) synchronized to the laser's Q‐switch. The ICCD camera settings—specifically, a 1 μs delay and a 5 μs gate—were optimized to maximize the signal‐to‐noise (S/N) ratio. The spectrometer featured a 600 L mm^−1^ grating and a 35 μm entrance slit, yielding a spectral resolution of ≈0.15 nm. The spectral window spanned from 249 to 333 nm, thereby accommodating the intense emission lines of both P (253.56 nm) and Ti (323.65 nm). Each recorded spectrum was generated from a single laser shot and thus corresponded to a single image pixel, without any data accumulation or averaging. Elemental intensities were extracted through a rigorous procedure that involved spectral line fitting, background subtraction, and subsequent summation of the background‐free intensities, culminating in the generation of a relative abundance map for each element.

## Conflict of Interest

The authors declare no conflict of interest.

## Supporting information

Supplementary Material

## Data Availability

The data that support the findings of this study are available from the corresponding author upon reasonable request.
