# Supplementary material for: Quantifying Titanium Exposure in Lung Tissues: A Novel Laser‐Induced Breakdown Spectroscopy Elemental Imaging‐Based Analytical Framework for Biomedical Applications
Source: Small Sci. 2024 Mar 3;4(5):2300307. doi: 10.1002/smsc.202300307 (PMC11935156; doi:10.1002/smsc.202300307)
Supplement: Supplementary file 1 — Supplementary Material [file SMSC-4-2300307-s001.pdf]

Supplementary Information

Title

Quantifying titanium exposure in lung tissues: a novel LIBS elemental imaging-based analytical framework for biomedical applications

*Vincent Gardette, Lucie Sancey, Marine Leprince, Laurent Gaté, Frederic Cosnier, Carole Seidel, Sarah Valentino, Frederic Pelascini, Jean-Luc Coll, Michel Péoc'h, Virginie Scolan, François Paysant, Vincent Bonneterre, Christophe Dujardin, Benoit Busser *, Vincent Motto-Ros*

This PDF file includes:

Figure S1. Quantitative LIBS analysis of select exogenous and endogenous elements in human FFPE lung specimens. (a) Hematoxylin and Eosin (H&E) staining of lung tissues from the same three autopsied individuals (Patients 1, 2, and 3), derived from 5 µm-thick tissue sections. Subsequent panels illustrate elemental LIBS imaging for (b) magnesium (Mg), (c) sodium (Na), (d) aluminum (Al), and (e) silicon (Si) in adjacent paraffin-embedded tissue blocks.

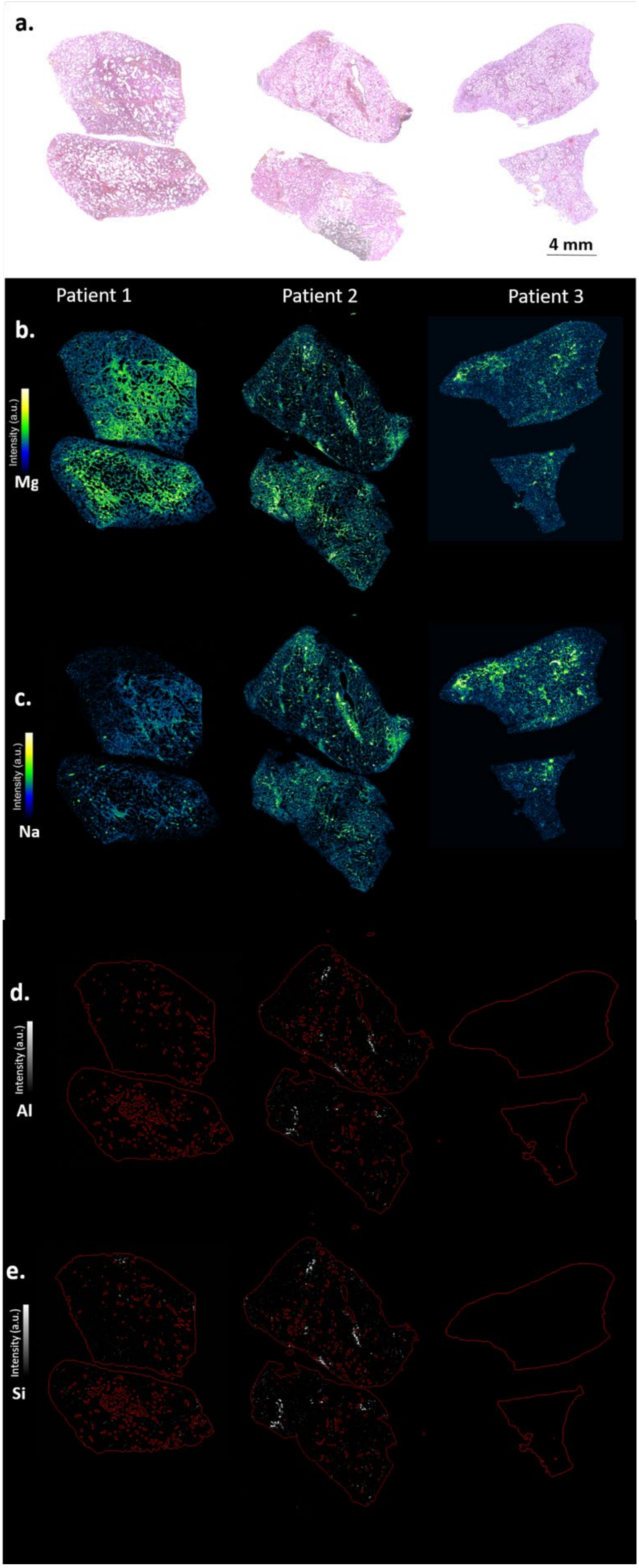
a.
4 mm
Patient 1
Patient 2
Patient 3
b.
Intensity (a.u.)
Mg
c.
Intensity (a.u.)
Na
d.
Intensity (a.u.)
Al
e.
Intensity (a.u.)
Si
